# 

**Published:** 1876

**Authors:** 


### TABLE OF CONTENTS.

ORIGINAL COMMUNICATIONS—

Opium in Pregnency and Parturition. By T. Curtis Smith, D.D., Ohio.................................................. 1
The Type of Diseases in Middle Georgia. By J. C. C. Blackburn, M.D., Barnesville, Ga................................................ 7
Sanguinaria Canandensis. By L. B. Anderson, Hanover county, Va.. 11
Gun Shot Wounds. By D. S. Handy, M.D., Stephenvill, Texas..... 15

SELECTIONS—

Electro-Therapeutics, etc. By Z. Collins McElroy, D.D., Zanesville, Ohio.................................................. 17
Dropsy of the Amnion. By W. H. Byford, A.M., D.D., Chicago College................................................ 29
Summary of Social and Scientific Progress in America.............. 34
Pueperal Insanity. By L. R. Landfear, M.D., Dayton, Ohio.......... 39
Chronic Albuminuria Treated with Iodide of Potassium. By T. S. Sharpe, D.D., Natchez, Miss.................................. 46
Hydrate of Chloral in Labor. By B. C. A. Prentiss, D.D., Greenfield, Missouri.............................................. 48

EXTRACTS AND GLEANINGS—

Cycilic Acid in Diptheria—Passage of a Worm through the Wall of the Abdomen—50. Treatment of Scarlatinal Albuminuria—51. Masturbation in Children—54. On the Local Use of Tannin—57. On Examination of the Heart—58. Researches of Dr. Curry, and Recent Views with Regard to Remedial Use of Water—59. Warburg's Tincture—60. Salicylic Acid—62. Tincture of Capsicum in the Treatment of "Tippling"—63. Bromide of Lithium—A Powder for the Treatment of Coryza—Sulpho-Carbolate of Sodium as a Prophylactie in Scarlatina—64. Nitrite of Amyl in Gastralgia—65. Obstinate Intermittent Fever Cured by Steam Bath—66. Carbolic Acid in Diabetis—A Paper on Rectified Spirit—67. The Form of Alcohol to be Used Medicinally—68. Chloroform and Morphine—69. Strychnia Poisoning—70. Chloral as a Local Application for Ulcers—71. The Communication of Tubercle in Food—73. The Use of Cold in Scarlatina—75. Sprain—76. Stomatitis Maserna—77. Duration of Bloodless Operations—Hydrastin in Gonorrhœa—78. Treatment for Hysterical Attacks—A New Salve for the Itch—79. Chinoidine Pills for Chills—Nitrite of Amyl in Hiccough—80. Collodion in Treatment of Carious Teeth—Use of Apocynum Cannabinum in Dropsies—Styptic Cotton in the Treatment of Otorrhœa—81. The Diagnosis of Secondary Heart Affections—82. The Catheter in the Enlarged Prostate—84. Puritus Vulva—The Cold Water Treatment—85. Administration of Raw Meat—Solubility of Borax in Glycerin—The Bromhydrate of Quinine—86. Sulphate of Cadnium in Acute Urethral Blenorrhagia—On the Use of Heat in Metorrhagia—Varix—87. The Treatment of Different Forms of Headache—88. Croton Chloral as a Nerve Sedative—Acidulated Gargles in Typhoid Fever—89. Cotton-Wool in the Ears as a Prophylactic and Curative Application in Coryza and Sore Throat—90. Cantharidal Strangury—Use of Electricity for Hyperprexia in Typhoid Fever—Syrup of Tolu—Neuralgia—91. Remedy for Chorea—Spinal Irritation—Treatment of Chorea by Arsenic in Large Doses—92. Hair Tonic—Enlarged Postate Mistaken for Calculus—93. Subcutaneous Injections of Stryconia for

Nervous Deafness—Carbolic Acid and Hyposulphites in Scarlatina—Digitalis in Bronchitis—94. A Hint in Giving Iodide of Potassium—Methods of Operating in Anal Fistula—Syrup of Coffee—Nitrate of Amyl in Neuralgic Affections—95. Eserine Disc in Tic—Novel Treatment of Diabetes Insipidus—Chloroform in Malaria—96. Treatment of Dyptheria—97. Treatment of Typhoid fever by the Cold Bath—98. Case of Rheumatic Hyperprexia Cured by one Cold Bath—100. Propylamin in Acute Rheumatism—101. Uterine Asthma —102. Alcohol in Glandular Swellings—103. Scrofulous Persons—Milk Diet—104. Boracic Acid in the Treatment of Ringworm—The Treatment of Vomiting in Pregnancy—105. Ganglion of the Wrist 106. Treatmeut of Tape-Worm by Creosote—A Surgical Nail—107. Antidote for Poisoning by Rhus Tuxicodendrum—Alcoholic Phthisis —108. Inunction—A Novel Expedient in a difficult Labor—109. Headaches, nervous—Action of Alkalies upon the Composition of the Blood—110. Chloroform in the Surgery of Infants—Treatment of Carbuncles by Strapping—Lime Glycerin for Burns—111. Dyptheria—Strychnia as an Anti-Emetic—Sore Nipples—Solution of Camphor in Erysipelas—112. Citric Acid in the Treatment of Cancer—Removal of Foreign Bodies From the Ear—Freckle Lotion—Treatment of Itch—Puritus Treated With Vinegar—Chloral in Nocturnal Emesis—113.

EDITORIAL AND MISCELLANEOUS—

Double Issue........ 113
A Word for THE MEDICAL RECORD........ 117
To Our Readers........ 113
From a Co-Laborer........ 119
Crying Evils Against the Profession........ 114
Our Book Table........ 121
Advertising Department, etc........ 120
Glad Tidings........ 114

